# Pet cats may shape the antibiotic resistome of their owner’s gut and living environment

**DOI:** 10.1186/s40168-023-01679-8

**Published:** 2023-10-23

**Authors:** Yiwen Yang, Xinwen Hu, Shuang Cai, Nan Hu, Yilin Yuan, Yinbao Wu, Yan Wang, Jiandui Mi, Xindi Liao

**Affiliations:** 1 Guangdong Provincial Key Laboratory of Agro-Animal Genomics and Molecular Breeding, Guangdong Laboratory of Lingnan Modern Agriculture, College of Animal Science, South China Agriculture University, Guangzhou, 510642 China; 2 Institute of Digestive Disease, Faculty of Medicine, Chinese University of Hong Kong, Hong Kong, 999077 China; 3 State Key Laboratory of Animal Nutrition, College of Animal Science and Technology, China Agricultural University, Beijing, 100193 China; 4 Department of Rehabilitation, The Second Affiliated Hospital of Guangzhou Medical University, Guangzhou, 510260 China; 5 State Key Laboratory of Veterinary Etiological Biology, College of Veterinary Medicine, Lanzhou University, Lanzhou, 730000 China

**Keywords:** Antibiotic resistome, Pet cat, Human, Gut, Living environment

## Abstract

**Background:**

Companion animals can contribute to the physical and mental health of people and often live in very close association with their owners. However, the antibiotic resistome carried by companion animals and the impact they have on their owners and living environment remain unclear. In this study, we compared the ARG profiles of cats, humans, and their living environments using metagenomic analysis to identify the core ARGs in the cat and human gut and explore the potential impact of cats on ARGs in the human gut through the environment.

**Results:**

Results showed that the abundance of ARGs in the cat gut was significantly higher than that in the human gut (*P* < 0.0001), with aminoglycoside and tetracycline resistance genes being the dominant ARGs in the cat gut. There was no significant difference in the abundance of total ARGs in the guts of cat owners and non-owners (*P* > 0.05). However, the abundance of aminoglycoside resistance genes including *APH(2'')-IIa* and *AAC(6')-Im* was significantly higher in cat owners than that in non-cat owners (*P* < 0.001). Also, ARG abundance was positively correlated with the frequency of cat activity in the living environment. *Enterobacteriaceae* was the dominant ARG host co-occurring in the cat gut, human gut, and living environment.

**Conclusions:**

Our results show that cats may shape the living environment resistome and thus the composition of some ARGs in the human gut, highlighting the importance of companion animal environment health.

Video Abstract

**Supplementary Information:**

The online version contains supplementary material available at 10.1186/s40168-023-01679-8.

## Background

Companion animals can contribute to the physical and mental health of people and often live in very close association with their owners, sometimes even sharing the same bed. However, the potential biosecurity safety risks associated with close contact between companion animals and their owners are often overlooked. Dogs and cats, by far the most popular companion animals, are important sources of zoonotic infections [1]. Studies have shown that pet dogs and cats can carry multiple human-associated pathogens and a variety of multi-drug-resistant bacteria, including methicillin-resistant *Staphylococcus aureus* and beta-lactam antibiotic-resistant *Enterobacteriaceae* [2, 3]. These antibiotic-resistant microorganisms may be transmitted from companion animals to humans through direct contact, ectoparasites, and aerosols. In particular, vulnerable populations, including immunocompromised elderly and infants, may be at greater risk. Moreover, as the number of companion animals continues to increase, the use of antibiotics in the clinical consultation of pets has become more common. Antibiotics that are not used appropriately could promote antibiotic resistance [4], emphasizing the fact that the risk of antibiotic resistance during companion animal husbandry cannot be ignored.

The effects of companion animals on the resistome of their owners and living environment are still unclear. Previous studies found that the likelihood of airborne transmission of antibiotic resistance genes (ARGs) was extremely low [5]. However, communication between cats and owners is more likely to be physical than airborne, thus the transmission route of ARGs between cats and owners is more likely to be via the living environment, such as working surfaces or the floor. In addition, there is currently a lot of research focused on ARG abundance changes and a lack of research on mobile-associated ARGs and pathogen-associated ARGs, even though these ARGs pose a greater threat to humans [6, 7].

In this study, we compared the ARG profile of cats, humans, and their living environments using metagenomic analysis to identify the core ARGs in the cat and human gut and explore the potential impact of cats on ARGs in the human gut through the environment.

## Materials and methods

### Experimental design and sample collection

**Fig. 1:**
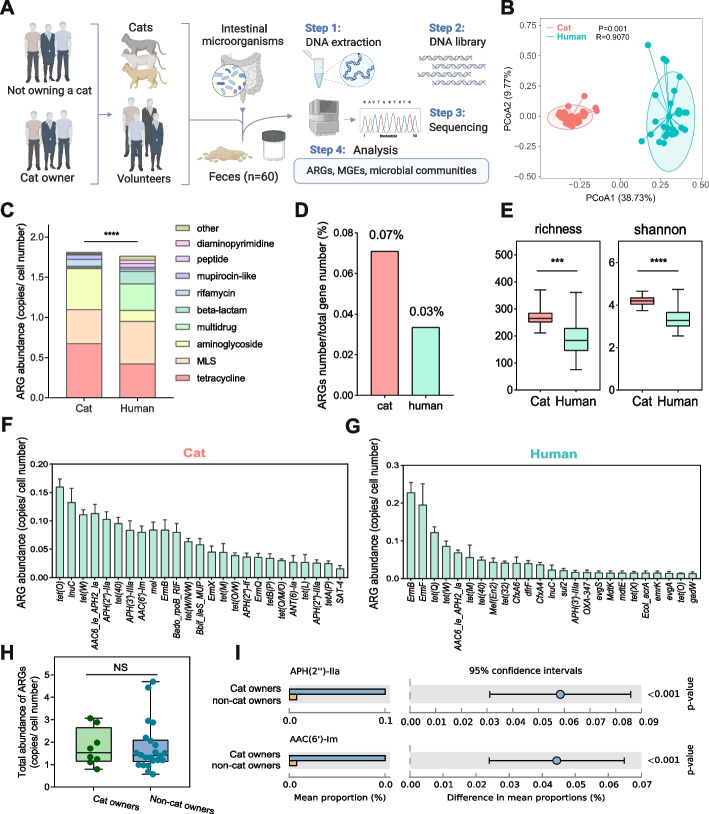
Comparison of resistome between cat gut and human gut. **A** Sampling flowchart; the **B** β-diversity, **C** abundance, **D** number, and **E** α-diversity of ARGs in cat and human gut. The core ARGs in **F** cat gut and **G** human gut. **H**, **I** ARG abundance in the gut of the cat owner and non-cat owners

To investigate the differences in ARG profiles from the intestines of pet cats and humans, 60 fecal samples were collected from 30 pet cats and 30 volunteers from Guangzhou, China, in August 2020 (Fig. 1A). Additional information about the cats and volunteers can be found in Supplementary Table S1 and Table S2. All fecal samples were collected with a sterile fecal collector, delivered to the laboratory within 4 h, and stored in a − 80 ℃ refrigerator for further use.

Next, the living environment of one of the cat owners was chosen (Sample ID: Human_4). And we collected samples from the cat-keeping environment, including areas such as the living room, dining room, balcony, small bedroom, medium bedroom, large bedroom, and cat litter (located in the toilet). The floor of each sampling point was wiped with a sterile swab in both horizontal and vertical directions. The homeowner cleaned the floor every 3 days, so we collected floor samples before each cleaning, a total of 5 times during April 2022. Ten swabs were collected at each sampling site each time and mixed into one sample, resulting in 35 mixed samples from the living environment. Finally, we downloaded 19 metagenomic sequencing samples from hospital environments from the National Center of Biotechnology Information (NCBI) website to compare the ARGs in the cat-keeping environment and the clinical environment.

### Metagenomic sequencing and microbial community analysis

Total DNA was extracted from samples using the QIAamp PowerFecal DNA Kit (Qiagen, Germany) following the manufacturer’s instructions. DNA samples were subjected to metagenomic sequencing at Novogene on the Illumina NovaSeq platform and 6 ~ 8 G of raw data were obtained for each sample. The metagenome raw data from all samples, including those from the hospital environment, were quality-controlled using KneadData (Trimmomatic 0.33) with default parameters (SLIDINGWINDOW:4:20 MINLEN:50) [8]. Subsequently, all human and cat DNA were identified with reference to the human (Homo_sapiens_Bowtie2_v0.1) and cat (GCF_018350175.1) genomes using bowtie2 software (–very-sensitive), and removed [9, 10]. The cleaned data of each sample was used for microbial community analysis based on the standard database of the Kraken2 software [11]. Finally, the clean data were assembled using Megahit v1.2.9 (based on the default kmer) [12]. These contigs were then subjected to open reading frames (ORFs) prediction using Prodigal software (v2.6.3). Thereafter the ORFs were clustered using Cd-hit (v4.8.1) with default parameters (-c 0.95). The ORFs were quantified using Salmon software (v1.4.0) [13–15].

### Antibiotic resistome analysis

The Comprehensive Antibiotic Resistance Database (CARD v3.2.5, https://card.mcmaster.ca/) was downloaded, collated, and used to build the ARG database using DIAMOND software (v2.0.15.153) [16]. Similarly, mobileOG-db (1.6 v1) was downloaded and used to build the mobile genetic element (MGE) database [17]. The ORFs files were compared with the ARG and MGE databases using DIAMOND software with the following parameters: *e* value 1e − 7, max-target-seqs 70, id 80. ARG-like-ORFs and MGE-like-ORFs were identified. The number of prokaryotic cells per sample was estimated using ARGs_OAP v3.2, and the abundance of ARGs and MGEs was corrected to copies/cell number [18].

### Identification of ARG hosts with MAG binning

The clean metagenome data of all samples were finely assembled using Metaspades (v3.14.0) with default parameters and the contigs were used to assemble metagenome-assemble genomes (MAGs) [19]. The MAGs were generated by Metabat2, Maxbins, and CONCOCT in the MetaWRAP pipeline [20]. Finally, MAGs were output based on > 50% completeness and < 10% contamination. Species classification of MAGs was performed using GTDB-TK v2.1.1 with the GTDB database (R207_v2) [21, 22]. To obtain ARG and MGE hosts, ARGs and MGEs in the MAGs were identified as in “Metagenomic sequencing and microbial community analysis” section. Virulence factors (VFs) in the MAGs were identified by comparing data to the VFDB_setA database (Last update: October 7, 2022), and these MAGs were considered bacterial pathogens in the gut of cats, humans as well as in the living environment.

### Identification of ARG public risks

The contigs from the metagenome assembly were compared with ARG, MGE, and VF databases using the BLAST tool to obtain the abundance of ARGs (ARGs-like contigs), mobile-associated ARGs (ARGs-MGEs like contigs) and mobile-associated ARGs in pathogenic bacteria (ARGs-MGEs-VFs like contigs) [7]. MetaCompare pipeline was then used to assess the resistome risk in the gut of cats and humans as well as in the hospital and living environment [6]. And MetaCHIP pipeline was used to assess the horizontal transfer risk of these ARGs [23].

### Data analysis and presentation

All data were first collated in WPS office 11.1.0 and significance analysis was performed using SPSS 22.0 (one-way ANOVA). And Tukey’s multiple comparisons test was used. Column graphs were plotted on GraphPad Prism 8.01, while box plots for gene and microbial analysis were drawn on STAMP 2.1.3. Principal coordinates analysis (PCoA) and redundancy analysis (RDA) were performed in R 4.2.2. Phylogenetic trees were drawn using iTOL (https://itol.embl.de/) [24]. Other images were generated in OriginPro 2023. Adobe Illustrator 22.1 was used for the graphic layout.

## Results

### Comparison of resistome in the gut of cats and humans

Results showed significant differences between the ARG β-diversity in the cat and human gut (*P* = 0.001) (Fig. 1B). The abundance of ARGs in the cat gut was 1.809 ± 0.070 copies/cell number, which was significantly higher than that in the human gut (1.765 ± 0.185 copies/cell number) (*P* < 0.0001) (Fig. 1C). The proportion of ARGs in the cat gut was 0.07% of the total number of genes, which was higher than that in the human gut (0.03%) (Fig. 1D). The Richness and Shannon index of ARGs in the cat gut was also higher than that in the human gut (Fig. 1E). The core ARGs in the cat gut were tetracycline, aminoglycoside, and MLS resistance genes, and the core ARGs in the human gut were tetracycline, MLS, and multidrug resistance genes. The abundance of tetracyclines (e.g., *tetO* and *teW*) and aminoglycoside resistance genes (e.g., *APH(2'')-IIa* and *ACC(6')-Im*) in the cat gut was significantly higher than that in the human gut (*P* < 0.05) (Fig. 1F, G and Figure S1). In addition, we found that although there was no significant difference between the total ARG abundance in the gut of the cat owner and non-cat owners (Fig. 1H), the abundance of ARGs such as *APH(2'')-IIa* and *ACC(6')-Im* were higher in the cat owner than those in non-cat owners (Fig. 1I). The abundance of these specific ARGs in the cat gut was also significantly higher than that in the human gut (*P* < 0.001).

### Risk of resistome transmission from cats to owner and living environment

**Fig. 2:**
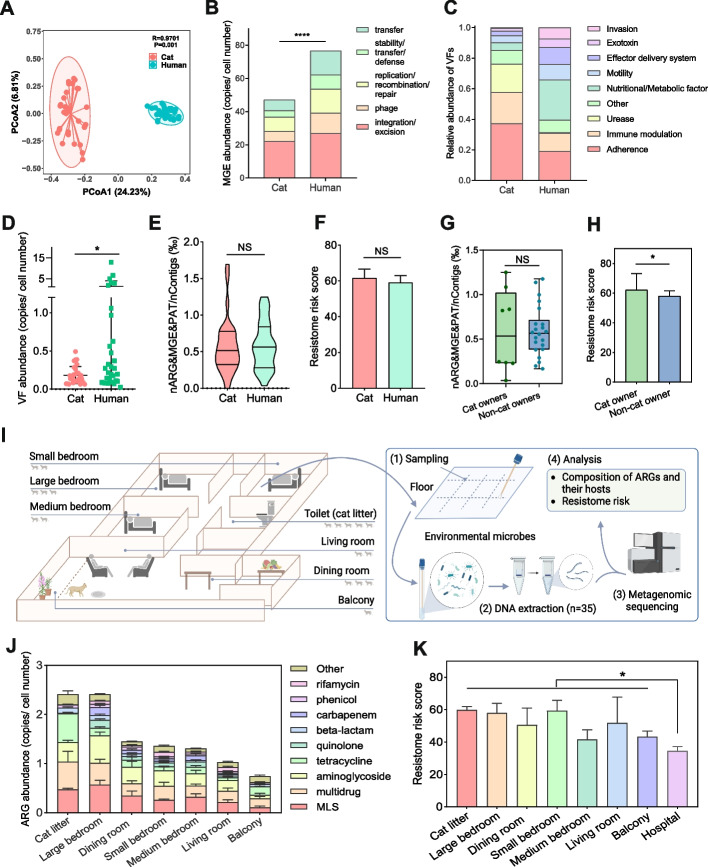
Resistome risk in cat, human, and their living environment. The **A** β-diversity and **B** abundance of MGEs in cat and human gut. **C**, **D** Abundance of VFs. **E** Proportion of pathogen-mobile-associated ARGs. **F** Resistome risk score in cat and human gut. **G** Proportion of pathogen-mobile-associated ARGs in the gut of the cat owner and non-cat owners. **H** Resistome risk score in cat and human gut in the gut of the cat owner and non-cat owners. **I** Sampling flowchart of the living environment. The more cats in the figure, the higher the frequency of cat activity. **J** ARG abundance and **K** resistome risk score in the living environment

Results from the MGEs composition analysis in the cat gut showed that the composition of MGEs in the cat gut and human gut differed significantly (*P* = 0.001) (Fig. 2A). The abundance of MGEs in the human gut was significantly higher than that in the cat gut (*P* < 0.0001), with 76.61 ± 3.28 copies/cell number and 47.03 ± 1.74 copies/cell number, respectively (Fig. 2B). The mobile-associated ARGs (contigs of ARGs-MGEs) in the human gut accounted for 0.59 ± 0.060% of the total contigs, which was significantly higher than the abundance in the cat gut (0.576 ± 0.068%) (*P* < 0.05) (Figure S2). The main types of VFs in the cat gut were adherence, immune modulation, and urease, while the main types of VFs in the human gut were adherence, nutritional/metabolic factors, and Immune modulation. The total abundance of VFs in the human gut was 1.319 ± 0.494 copies/cell number, which was significantly higher than that in the cat gut (0.183 ± 0.021 copies/cell number) (*P* < 0.05) (Fig. 2C, D). However, there was no significant difference in the proportion of pathogen-mobile-associated ARGs (contigs of ARGs-MGEs-VFs) in the cat gut and the human gut. We also found a non-significant difference in resistome risk scores between the human and cat gut (Fig. 2E, F).

In addition, there was no significant difference in the proportion of pathogen-mobile-associated ARGs (contigs of ARGs-MGEs-VFs) in the guts of the cat owner and the non-cat owner, but the resistome risk score in the cat owner’s gut was significantly higher than that of the non-cat owner’s gut (*P* < 0.05) (Fig. 2G, H). These suggest that cat-derived ARGs have a risk of affecting the resistome in the human gut. We collected samples from the cat-keeping environment for the study (Fig. 2I). In this study, the large room was the most active area for cat, followed by the dining room, small room, medium room living room, and the balcony. For the living environment analysis, we found the highest abundance of ARGs in the cat litter and the large bedroom with 2.414 ± 0.287 copies/cell number and 2.411 ± 0.330 copies/cell number, respectively, followed by the dining room, small bedroom, medium bedroom and living room (Fig. 2I, J). The lowest abundance of ARGs was found on the balcony with 0.744 ± 0.104 copies/cell number. The abundance of ARGs in the living environment was positively correlated with the frequency of cat activity. This suggests a possible transmission of ARGs from the cat gut to the living environment, which in turn affects the owner. Rather surprisingly, the risk of antibiotic resistome transfer was higher in the living environment than in the hospital (Fig. 2K). reminding us of the importance of focusing on the resistome risk in the keeping-cat living environment.

### Host composition characteristics and horizontal transfer risk of ARG

**Fig. 3:**
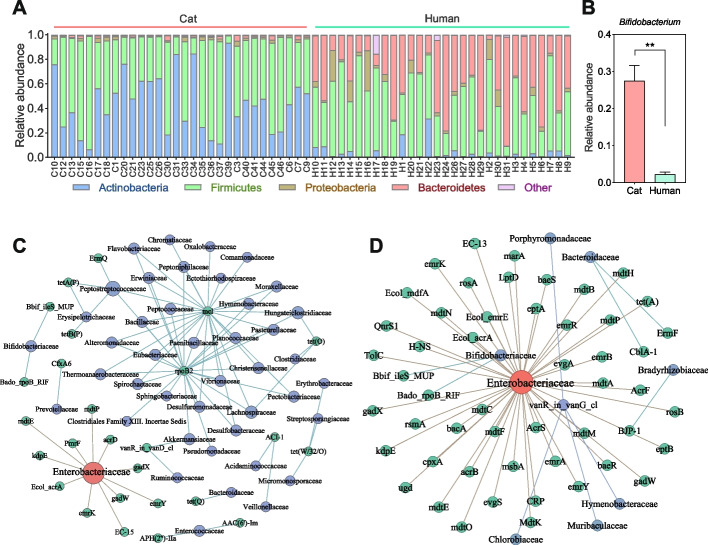
Bacterial community compositions in the cat and human gut. Relative abundance of **A** top 4 phyla and **B** *Bifidobacterium*; co-occurrence pattern of ARGs and bacterial communities in **C** cat gut and **D** human gut

There were significant differences in the bacterial community composition between the cat and human gut, with the highest relative abundance of Actinobacteria (0.45 ± 0.24) and Firmicutes (0.52 ± 0.23) in the cat gut and Firmicutes (0.49 ± 0.20) and Bacteroidetes (0.40 ± 0.23) in the human gut (Fig. 3A). In addition, the abundance of *Bifidobacterium* in the cat gut was significantly higher than that in the human gut, and the abundance of *Bifidobacterium* was higher in the gut of the cat owner compared to non-cat owners (Fig. 3B and Supplementary Figure S3). This suggested that cat microbes may influence the microbial composition of the human gut, perhaps including ARG hosts.

**Fig. 4:**
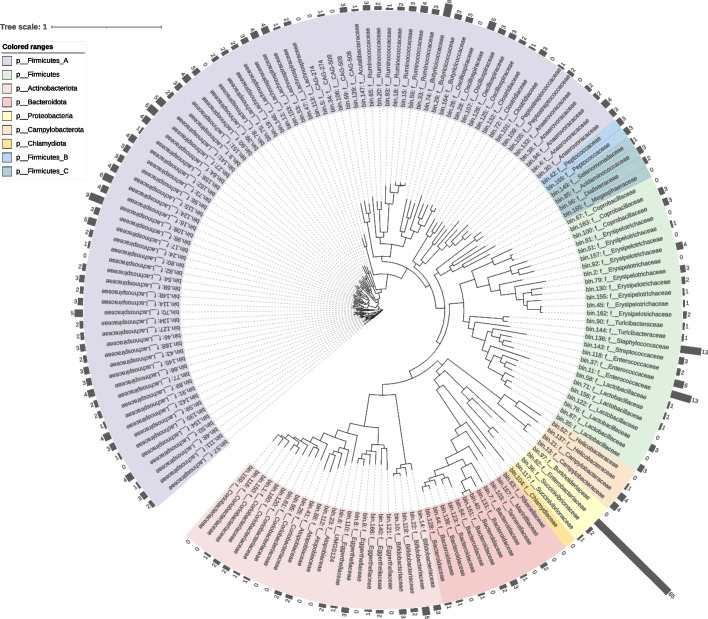
The number of ARGs carried by MAGs in cat gut

**Fig. 5:**
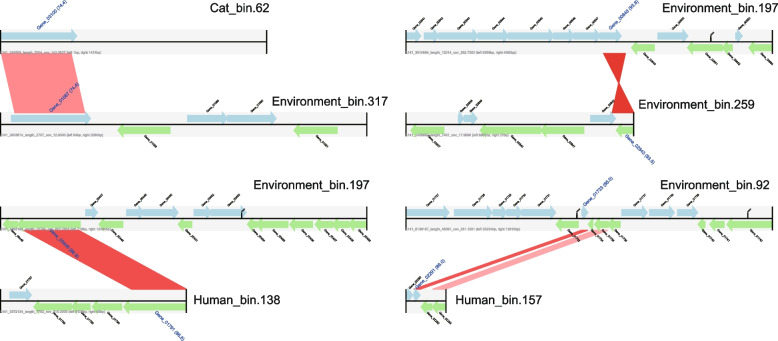
Horizontal gene transfer occurring in MAGs from cat gut, human gut, and environment

We mined for ARG hosts using co-occurrence analysis and found that cats and humans share a common host in Enterobacteriaceae, which was significantly and positively correlated with 12 and 46 ARGs (ρ > 0.6, *P* < 0.01), respectively (Fig. 3C, D). To further identify the major ARG host in the cat and human gut, we then used a metagenome assembly to obtain 828 MAGs, 161 from the cat gut, 313 from the human gut, and 351 from the living environment, composed mainly of Firmicutes, Actinobacteriota, Bacteroidota, and Proteobacteria (Figure S4). Most of the bacteria at different phylum levels identified from cat and human feces were found to carry ARGs, suggesting that ARGs are prevalent in environmental bacteria (Fig. 4 and Figure S5). The Enterobacteriaceae were found to be the major ARG hosts in the cat and human gut, being detected to carry 65 (bin. 62) and 73 (bin. 157) ARGs. More worryingly, the Enterobacteriaceae includes common pathogenic bacteria such as *Escherichia*, *Salmonella*, and *Shigella*, which increase the risk of antibiotic resistance. Further horizontal transfer assessment of these MAGs revealed that bin62 (Enterobacteriaceae), the main ARG host in the cat gut, is at risk of gene transfer to living environmental bin317 (Fig. 5). There was also a risk of horizontal transfer between different MAGs in the living environment, such as between bin197 and bin259. Finally, we identified a risk of horizontal transfer of bacterial genes from the living environment to the human gut, such as between bin197 and bin92 in the environment, and bin138 and bin157 in the human gut. Among them, bin157 was the main ARG host in the human gut.

## Discussion

### Companion animals shape the resistome in the human gut

The intestinal tract contains a large number of microorganisms that make up a relatively constant resident bacteria and transient bacteria that come in from the external environment [25]. Resident bacteria are the main components of the intestinal flora and maintain a dynamic balance with the environment and the host by promoting normal physiological functions, such as nutrition, immunity, and digestion in the host [26]. Transient bacteria are harmless or pathogenic foreign bacteria that enter the intestinal tract through contact and aerosols. Normally, resident bacteria adheres, colonizes, and multiplies in the intestine, forming a barrier that inhibits and rejects the transient bacteria through antagonism, thereby protecting intestinal homeostasis and host health. However, through prolonged exposure, microorganisms in the living or working environment also shape the intestinal flora, and changes in the composition of microorganisms, which are important hosts for ARGs, can lead to changes in the resistome [27].

In this study, we found that the composition and diversity of the bacterial community in the cat gut differed significantly from those in the human gut. Also, the α- and β-diversity of ARGs in the cat gut differed significantly from those in the human gut, and the abundance of ARGs in the cat gut was significantly higher than that in the human gut, including all types of ARGs. Bacteria carrying a high risk of *mcr-1* resistance gene have also been isolated from cats and dogs [28]. Therefore, the higher risk of antibiotic resistance present in the cat gut cannot be ignored. In addition, this study found that the abundance of *APH(2''-IIa* and *ACC(6')-Im* resistance genes in the gut was significantly higher in the cat owner than in non-cat owners. These resistance genes are also core ARGs in the cats tested here. Previous studies have also shown a strong correlation in macrolide resistance genes between pet dogs and their owners [29]. Belas et al. (2020) found that the resistome of companion animals is similar to those of people in close contact with them [30]. Thus, it can be assumed that companion animals shape the gut resistome of their owners to a certain extent.

We also showed that the core ARG hosts in both the cat gut and human gut were Enterobacteriaceae and a risk of horizontal gene transfer between these core ARG hosts and different bacteria in the living environment and human gut. It can be hypothesized that these core ARG hosts in the feline gut can enter the human gut through different pathways as transient bacteria and further alter the resistome of the owner’s gut through horizontal gene transfer. Of course, it is also possible that these ARGs and their hosts may attach to and colonize the human intestine over longer periods. Studies have shown that the gut microbiome and resistome of feeders working on farms for long periods can be reshaped [31, 32]. More worryingly, the core ARG hosts identified in this study, Enterobacteriaceae, also include pathogenic bacteria such as *Escherichia*, *Salmonella*, and *Shigella*, which may pose a more complex risk of antibiotic resistance and pathogenicity.

It is very unfortunate that only 30 volunteers and 30 cats were sampled in the first part of the trial in this study. Our original plan was to collect more than 100 volunteer samples, but COVID-19 pandemic outbreak suddenly became severe and some volunteers had to withdraw from our study. Although the study did not go as smoothly as expected, the results obtained have important reference value.

### Pathways by which companion animals influence the human resistome

The main route of transmission of ARGs and their hosts in the environment is through contact, such as air and surfaces [33]. It has been suggested that air is an important pathway for companion animals to influence the human resistome. Because of the distinctive smell in cat-feeding households, it was speculated that antibiotic-resistant microorganisms and ARGs volatilized from cats might be floating in the air of cat-feeding environments. Besides, previous studies have also confirmed that microorganisms and ARGs in the environment can enter humans in the form of aerosols [34, 35]. Surprisingly, our results showed that the microorganisms and ARGs volatilized from the cat gut (feces) into the air were minimal, suggesting that air is not the main route for the transmission of ARGs from cats to owners [5]. Pet cats are curious animals and will cover almost the entire living space in their range of activities. Therefore, we collected floor samples from various areas of the living environment and found that the abundance of ARGs in these areas was positively correlated with the frequency of pet cat activity. Horizontal gene transfer was found to occur between the cat gut, the environment, and the human gut, making it feasible that pet cats can affect the human resistome through their activities in the living environment, and that this effect is greater than the airborne effect. This is especially true for rooms where cats are more active.

Due to homeostatic regulation of the intestine [36], microorganisms that enter the human gut from the cat and the living environment, including ARG hosts, are normally transient for the human gut and rarely attach to and colonize the gut. We found a higher abundance of MGEs in the human gut samples than in the cat and the environmental samples, including phage, integron, and transposon types. These MGEs act as important factors in the horizontal gene transfer of ARGs in different microorganisms [36]. This suggests that there is a greater risk of ARG horizontal gene transfer in the human gut. Also, horizontal gene transfer is an important influencing factor for environmental resistome variation, with MGEs explaining up to 10.3% of the ARG variation in wastewater; 13.9% of the ARG variation in pig intestines, and 13.76% of the ARG variation in dog intestines [29, 37, 38]. Additionally, we then analyzed the contigs of ARGs and MGEs and found that the proportion of the mobile ARGs (MGE-ARG-like contigs) in the cat gut and the human gut was 0.576 ± 0.068‰ and 0.590 ± 0.060‰ of the total contigs, respectively. Horizontal gene transfer was also found between different microorganisms in cat feces, the living environment, and the human gut.

**Fig. 6:**
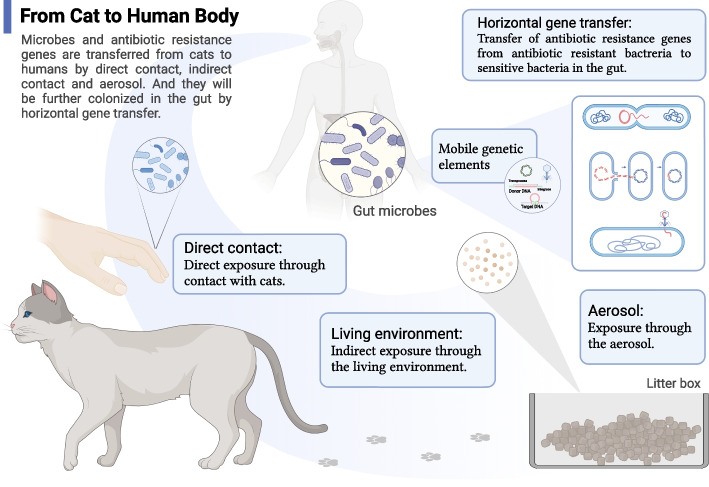
The formation of resistance from the cat to the human gut and living environment

In summary, we mapped the shaping process of the resistome from the cat to the human gut and living environment (Fig. 6). The ARGs and their hosts in cats are brought to the living environment through the activity of the cat, shaping the resistome in the environment, which in turn affects the human gut resistome. There are also a small number of ARG host that enter the human body through the respiratory tract via aerosols [5]. Of course, direct contact with cats may be the main route through which ARG hosts enter the human gut [39]. The ARGs that enter the human gut are then repositioned in the intestine, such as by horizontal gene transfer, to shape the resistome of the owner.

### The resistome risk from companion animals is low

ARGs are commonly found in natural environments, even in extreme environments, such as the deep-sea and Arctic permafrost [40, 41]. Therefore, the presence of ARGs or the presence of the horizontal spread of ARGs should not be considered a high resistome risk. There are several methods with which to assess the resistome risk of the environment. The most direct approach is to detect the abundance of ARG score in the environment, both in absolute and normal abundance. It is believed that the higher the abundance of ARGs in the environment, the higher the resistome risk. Resistome risk could also be estimated by combining the analysis of the abundance of total ARGs, mobile ARGs (MGEs-ARGs like contigs), and mobile ARGs associated with pathogenic bacteria (MGEs-VFs-ARGs like contigs) [6, 7]. In this study, we used both methods to assess resistome risk in cat-keeping households and found no significant differences in the abundance of ARGs in cat owners and non-cat owners, as well as no significant differences in the resistome risk scores in the human and cat gut. In addition, we found that the abundance of *Bifidobacterium*, which is considered to be a beneficial bacterium, was significantly higher in the cat gut than that in the human gut. Also, the abundance of *Bifidobacterium* in the gut of the cat owner was significantly higher than that of non-cat owners. This evidence suggests that cat ownership can shape the resistome in the owner's gut and living environment, but that the resistome risk is small. Additionally, we could not find any reports of antibiotic-resistant contamination due to pet ownership. Despite this, cleanliness is important when keeping cats. In addition, ARGs can be reduced by adjusting the cat’s diet and the rational use of antibiotics for pets to prevent ARG enrichment.

## Conclusion

We found that the ARG abundance in the cat gut was significantly higher than that in the human gut, the resistome risk in the gut of the cat owner was higher than that in non-cat owners, and the ARG abundance in the living environment was positively correlated with the frequency of cat activity. We also showed that the cat gut and the human gut share the same core ARG hosts (Enterobacteriaceae). These results suggest that pet cat may shape the antibiotic resistome in human gut. And the horizontal gene transfer between bacteria in the environment as well as in the human gut can further contribute to resistome shaping. We are reminded of the resistome risk during companion animal keeping and to minimize the transmission pathways of companion animal-derived resistance by managing the living environment and ensuring animal health.

### Supplementary Information

**Additional file 1:** **Table S1.** Information about the cats in this study. **Table S2.** Information about the volunteers in this study. **Figure S1.** The core ARGs in (A) cat gut and (B) human gut. **Figure S2.** Relative abundance of the mobile-associated ARGs. **Figure S2.** Relative abundance of the mobile-associated ARGs. **Figure S4.** Relative abundance of the MAGs from cat gut, human gut and living environment. **Figure S5.** ARGs abundance in MAGs from the human gut.

## Data Availability

All sequencing data for this study are available from the NCBI database (Accession number: PRJNA944553). The accession number for the hospital environmental metagenome sequencing data used in this study is PRJNA726763.
