# Supplementary material for: Pet cats may shape the antibiotic resistome of their owner’s gut and living environment
Source: Microbiome. 2023 Oct 23;11:235. doi: 10.1186/s40168-023-01679-8 (PMC10591416; doi:10.1186/s40168-023-01679-8)
Supplement: Supplementary file 2 — Additional file 1: Table S1. Information about the cats in this study. Table S2. Information about the volunteers in this study. Figure S1. The core ARGs in (A) cat gut and (B) human gut. Figure S2. Relative abundance of the mobile-associated ARGs. Figure S2. Relative abundance of the mobile-associated ARGs. Figure S4. Relative abundance of the MAGs from cat gut, human gut and living environment. Figure S5. ARGs abundance in MAGs from the human gut. [file 40168_2023_1679_MOESM1_ESM.docx]

Supplementary Material for

**Pet cats may shape the antibiotic resistome of their owner’s gut and living environment**

Yiwen Yang^1^*, Xinwen Hu^2^, Shuang Cai^3^, Nan Hu^4^, Yilin Yuan^1^, Yinbao Wu^1^, Yan Wang^1^, Jiandui Mi^5^, Xindi Liao^1^*

^1^ Guangdong Provincial Key Laboratory of Agro-Animal Genomics and Molecular Breeding, Guangdong Laboratory of Lingnan Modern Agriculture, College of Animal Science, South China Agriculture University, Guangzhou 510642, China

^2^ Institute of Digestive Disease, Faculty of Medicine, Chinese University of Hong Kong, Hong Kong 999077, China

^3^ State Key Laboratory of Animal Nutrition, College of Animal Science and Technology, China Agricultural University, Beijing 100193, China

^4^ Department of Rehabilitation, The Second Affiliated Hospital of Guangzhou Medical University, Guangzhou 510260, China

^5^ State Key Laboratory of Veterinary Etiological Biology, College of Veterinary Medicine, Lanzhou University, Lanzhou 730000, China

***Corresponding author:**

Yiwen Yang, Ph.D.

E-mail: yiweny@foxmail.com

Xindi Liao, Ph.D.

E-mail: xdliao@scau.edu.cn

**Table S1.** Information about the cats in this study

| **Sample ID** | | **Collection date** | **Breed** | **Gender** | **Age/days** | **Other** |
| --- | --- | --- | --- | --- | --- | --- |
| Cat_1 | 2020.8.12 | | British Shorthair | Female | 90 | Kitten |
| Cat_3 | 2020.8.16 | | British Shorthair | Female | 540 | Adult |
| Cat_6 | 2020.8.12 | | British Shorthair | Male | 120 | Kitten |
| Cat_7 | 2020.8.16 | | British Shorthair | Male | 150 | Kitten |
| Cat_9 | 2020.8.16 | | British Shorthair | Female | 180 | Kitten |
| Cat_10 | 2020.8.16 | | British Shorthair | Male | 180 | Kitten |
| Cat_12 | 2020.8.30 | | British Shorthair | Male | 730 | Adult |
| Cat_13 | 2020.8.31 | | Ragdoll | Male | 365 | Adult |
| Cat_15 | 2020.8.29 | | Ragdoll | Male | 60 | Kitten |
| Cat_16 | 2020.8.29 | | British Shorthair | Male | 90 | Kitten |
| Cat_17 | 2020.8.21 | | American Shorthair | Male | 2190 | Adult |
| Cat_18 | 2020.3.30 | | Munchkin | UK | 2095 | Adult |
| Cat_20 | 2020.8.10 | | Ragdoll mixes | Male | 180 | Kitten |
| Cat_21 | 2020.8.9 | | British Shorthair | Male | 75 | Kitten |
| Cat_23 | 2020.8.9 | | British Shorthair | Female | 180 | Kitten |
| Cat_25 | 2020.8.9 | | British Shorthair | Female | 150 | Kitten |
| Cat_26 | 2020.8.9 | | Ragdoll | Female | 730 | Adult |
| Cat_30 | 2020.8.4 | | Scottish Fold | Male | 120 | Kitten |
| Cat_31 | 2020.8.4 | | British Shorthair | Female | 730 | Adult |
| Cat_33 | 2020.8.4 | | British Shorthair | Female | 150 | Kitten |
| Cat_34 | 2020.8.3 | | British Shorthair | Female | 600 | Adult |
| Cat_35 | 2020.8.4 | | British Shorthair | Female | 90 | Kitten |
| Cat_36 | 2020.8.4 | | British Shorthair | Female | 90 | Kitten |
| Cat_37 | 2020.8.4 | | British Shorthair | Male | 120 | Kitten |
| Cat_39 | 2020.8.4 | | British Shorthair | Male | 120 | Kitten |
| Cat_40 | 2020.8.4 | | British Shorthair | Female | 120 | Kitten |
| Cat_41 | 2020.8.4 | | British Shorthair | Male | 120 | Kitten |
| Cat_44 | 2020.8.4 | | British Shorthair | Male | 365 | Adult |
| Cat_45 | 2020.8.4 | | British Shorthair | Female | 120 | Kitten |
| Cat_46 | 2020.8.4 | | British Shorthair | Female | 120 | Kitten |

**Table S2.** Information about the volunteers in this study

| **SampleID** | **Gender** | **Age/years** | **Cat owner** | **Cat** |
| --- | --- | --- | --- | --- |
| Human_1 | Male | 27 | Yes | Cat_1 |
| Human_2 | Male | 27 | No | - |
| Human_3 | Female | 26 | No | - |
| Human_4 | Female | 25 | Yes | Cat_2 |
| Human_5 | Female | 26 | No | - |
| Human_6 | Male | 25 | No | - |
| Human_7 | Female | 27 | No | - |
| Human_8 | Male | 26 | No | - |
| Human_9 | Male | 25 | No | - |
| Human_10 | Female | 25 | No | - |
| Human_11 | Female | 26 | No | - |
| Human_12 | Male | 26 | No | - |
| Human_13 | Male | 26 | No | - |
| Human_14 | Male | 24 | No | - |
| Human_15 | Male | 26 | Yes | Cat_3 |
| Human_16 | Male | 25 | No |  |
| Human_17 | Female | 26 | No |  |
| Human_18 | Male | 25 | No |  |
| Human_19 | Male | 30 | Yes | Cat_4 |
| Human_20 | Male | 28 | No |  |
| Human_21 | Female | 28 | No |  |
| Human_22 | Male | 28 | Yes | Cat_5 |
| Human_23 | Male | 28 | Yes | Cat_6 |
| Human_24 | Male | 28 | No |  |
| Human_26 | Female | 27 | No |  |
| Human_27 | Female | 26 | No |  |
| Human_28 | Female | 26 | No |  |
| Human_29 | Male | 25 | Yes | Cat_7 |
| Human_30 | Female | 27 | Yes | Cat_8 |
| Human_31 | Female | 28 | No |  |


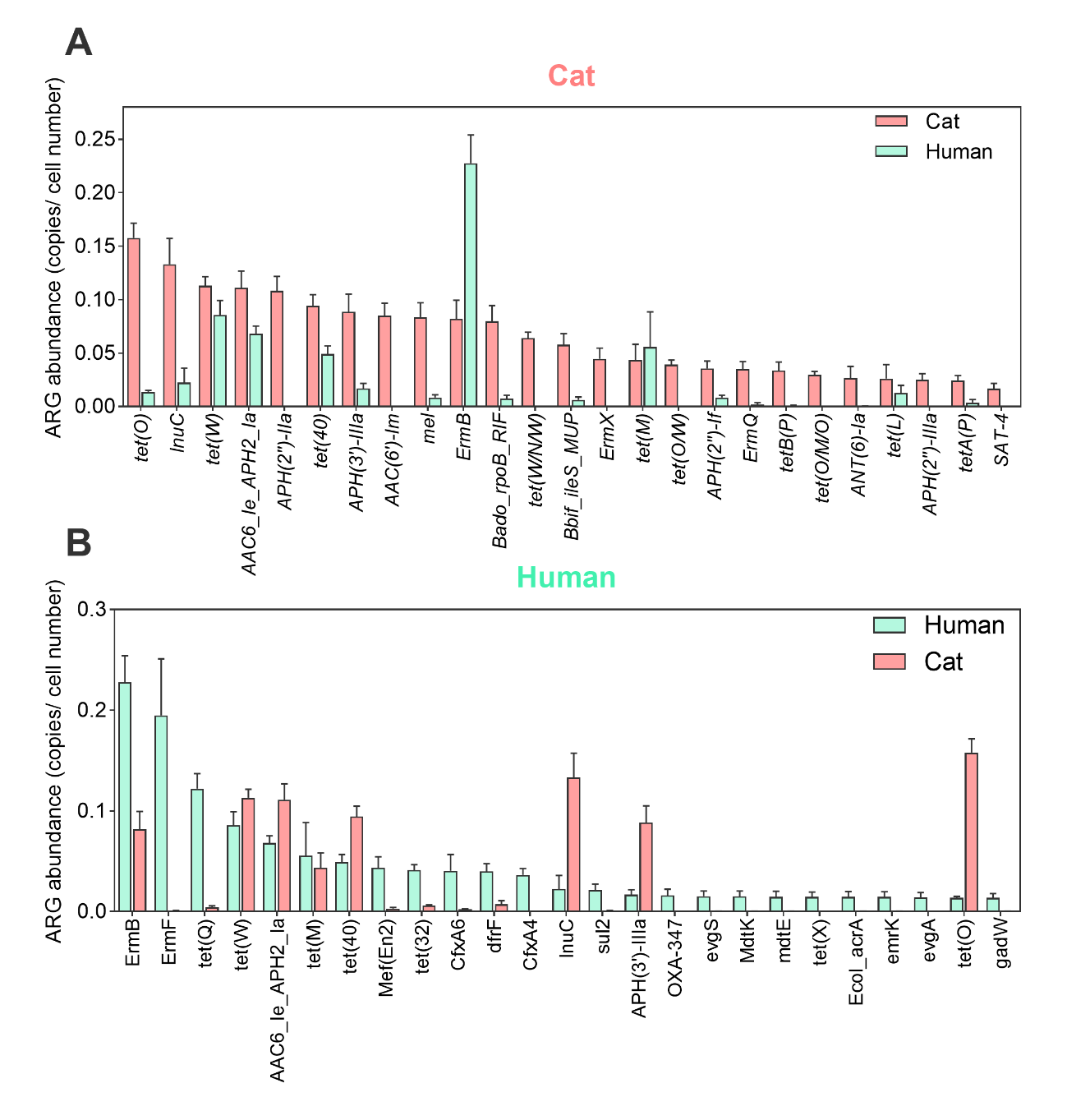


Figure S1. The core ARGs in (A) cat gut and (B) human gut.


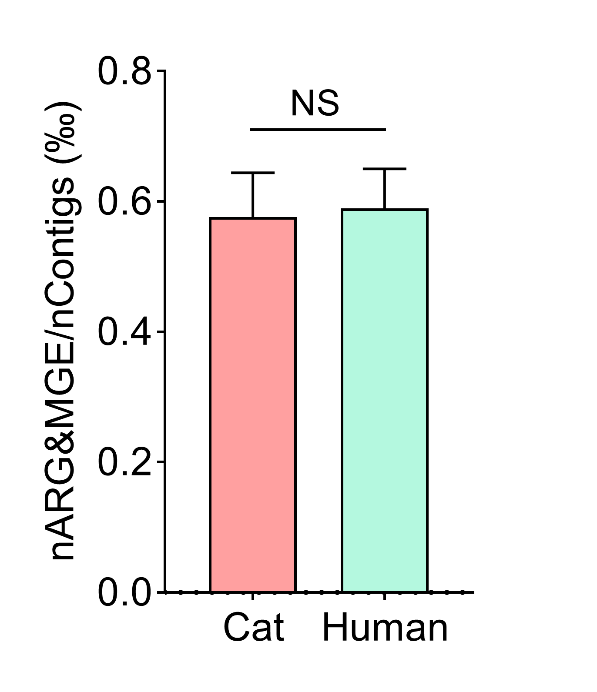


Figure S2. Relative abundance of the mobile-associated ARGs


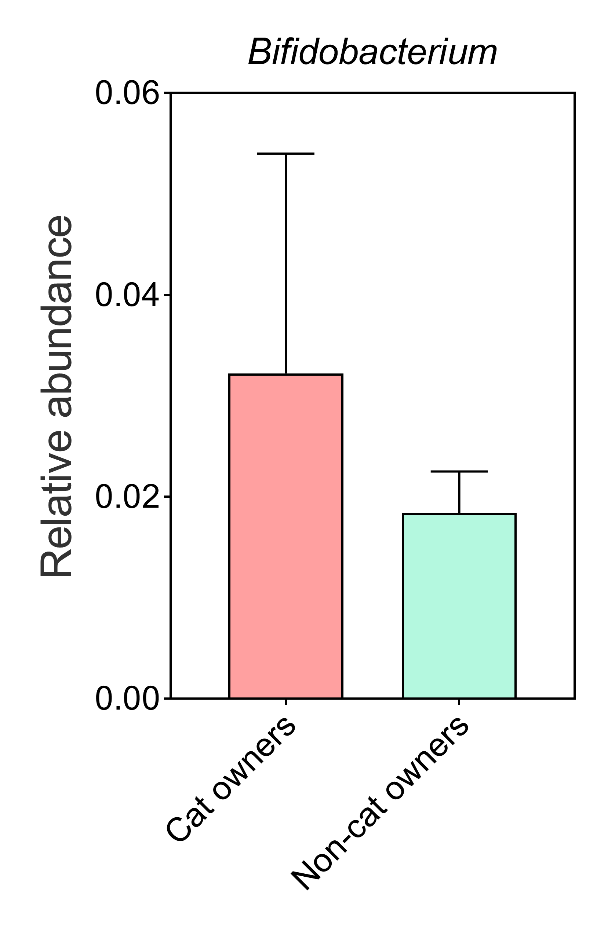


Figure S3. Relative abundance of *Bifidobacterium*


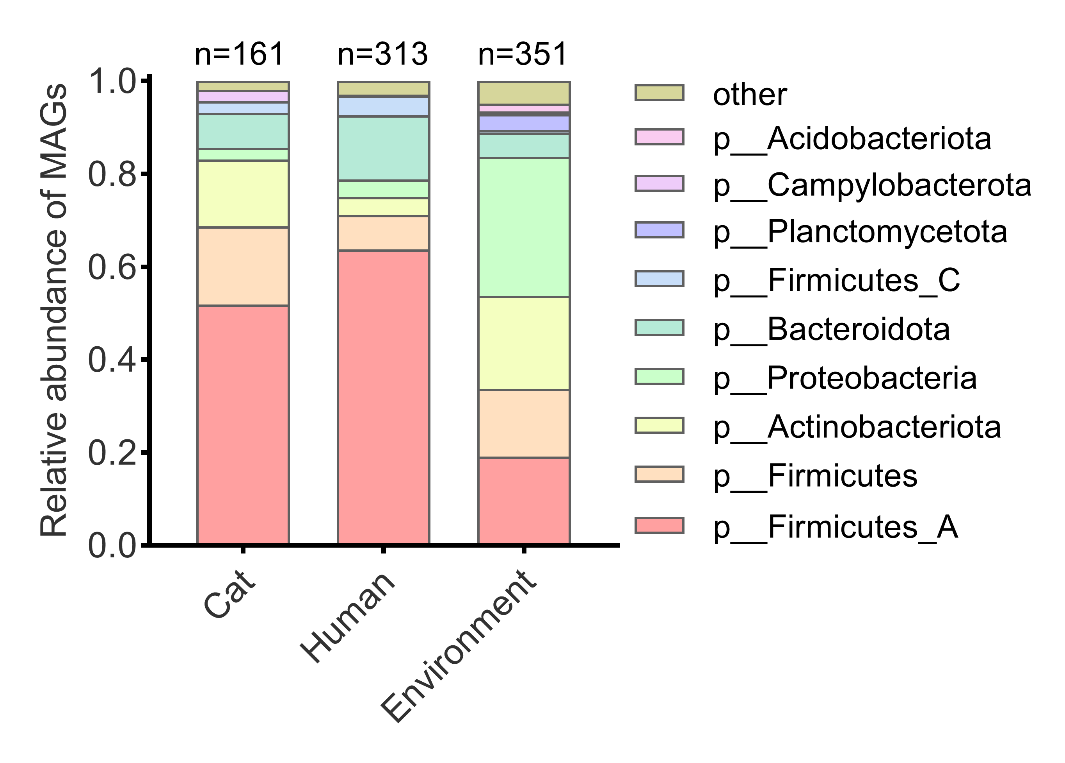


Figure S4. Relative abundance of the MAGs from cat gut, human gut and living environment


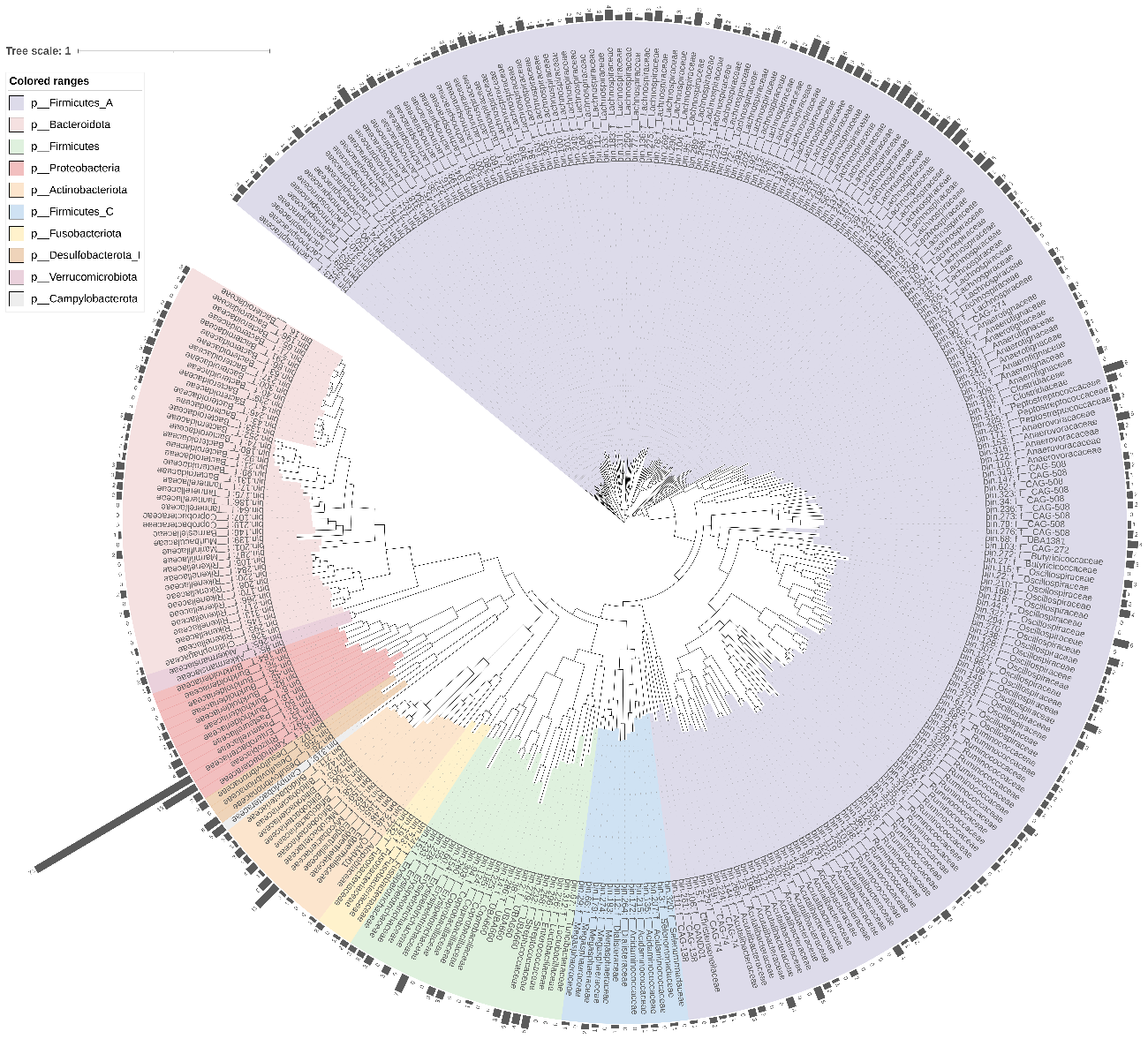


Figure S5. ARGs abundance in MAGs from the human gut
